# Mendelian randomization study of circulating leukocytes counts reveals causal associations with inflammatory bowel disease

**DOI:** 10.1097/MD.0000000000041969

**Published:** 2025-03-28

**Authors:** Chengtao Liang, Zhibin Wang, Yuhe Mai, Junxiang Li, Qiuhong Dai, Yali Yuan, Muyuan Wang, Yuyue Liu, Wenji Zhang, Yitong Li, Xinyu Lu, Zhengdao Lin, Tangyou Mao

**Affiliations:** a Dongfang Hospital, Beijing University of Chinese Medicine, Beijing, PR China; b Qinhuangdao Hospital of Traditional Chinese Medicine, Qinhuangdao, PR China.

**Keywords:** causal associations, circulating leukocytes counts, inflammatory bowel disease, Mendelian randomization

## Abstract

Inflammatory bowel disease (IBD) is a chronic recurrent IBD, whose cause involves the interaction between genetic and environmental factors. Although there is a recognized link between immune response and IBD, the causal relationship between circulating immune cell counts and IBD remains controversial. This study aimed to elucidate the causal relationship between genetically predicted circulating immune cell counts and IBD. We conducted a bidirectional 2-sample Mendelian randomization (MR) study using aggregated statistics from genome-wide association studies. The causal relationship between 5 circulating leukocytes cells (monocytes, lymphocytes, eosinophils, basophils and neutrophils) counts and IBD, including ulcerative colitis (UC) and Crohn disease (CD) was analyzed. Horizontal pleiotropy test and heterogeneity test were used to ensure the stability of the results. Our findings indicated that monocytes, lymphocytes, eosinophils, and basophils count were not significantly associated with IBD, however, elevated circulating neutrophils count was significantly associated with higher risk of IBD [odds ratio (OR) = 1.0017; 95% confidence interval (CI) = 1.0004–1.003; *P* = .009] and UC [OR = 2.465; 95% CI = 1.236–4.916; *P* = .01]. In addition, we also found that IBD [OR: 12.07; 95% CI = 1.909–76.316; *P* = .008] and CD [OR = 1.014; 95% CI = 1.004–1.023; *P* = .005] were significantly associated with higher circulating neutrophils count in reverse MR. This MR study provides genetic evidence for the causal relationship between the genetically predicted increase in circulating neutrophils count and the risk of IBD (UC and CD). This finding stresses the need for further exploring physiological functions of neutrophils in order to develop effective strategies against IBD.

## 1. Introduction

As a chronic recurrent inflammatory disease of the gastrointestinal tract, inflammatory bowel disease (IBD) could occur in any part of the gastrointestinal tract and mainly includes ulcerative colitis (UC) and Crohn disease (CD). The number of patients with IBD is on the rise, with nearly 3.9 million women and 3 million men affected worldwide,^[1]^ and the global prevalence of IBD has been increasing with the improvement of living standards.^[2]^ At present, the pathogenesis of IBD has not been fully understood. According to existing research, it is mainly related to the interaction between genetics and environmental factors,^[3]^ mainly caused by an inflammatory response from a genetically susceptible host to gut microbes.^[4]^

Circulating leukocytes are spherical blood cells, and the total number in normal adults is (4.0–10.0) × 10^9^/L, which could be divided into 5 major circulating leukocytes subtypes, including monocytes, lymphocytes, eosinophils, basophils and neutrophils according to different cell morphology and function. Leukocytes could pass through the capillary wall through deformation to concentrate on the site of bacterial invasion, and mainly play a protective role on host healthy. Previous studies suggested that improper migration and adhesion of leukocytes were signs of chronic inflammation, contributing to the occurrence and development of IBD.^[5,6]^ Therefore, IBD is mainly characterized by a large number of circulating leukocytes infiltrating into the inflamed intestinal mucosa,^[7]^ so blocking leukocytes transport is an essential strategy for treating IBD.^[8]^ In recent years, the relationship between circulating leukocytes and IBD has been thoroughly investigated, however, the majority of these studies have been observational in nature, which means that they are constrained by inherent issues such as reverse causality and residual confounding.^[9]^ Therefore, the causal relationship between circulating immune cell counts and IBD needs to be further explored. Mendelian randomization (MR) is an emerging statistical method for analysis using aggregated genetic data,^[10]^ which could be used to assess causality in associations between observed exposures and clinically relevant outcomes.^[11]^ Therefore, we used MR to conduct causal analysis from a genetic perspective to explore the causal relationship between circulating leukocytes and IBD.

## 2. Materials and methods

### 2.1. Study design

**Figure 1.**
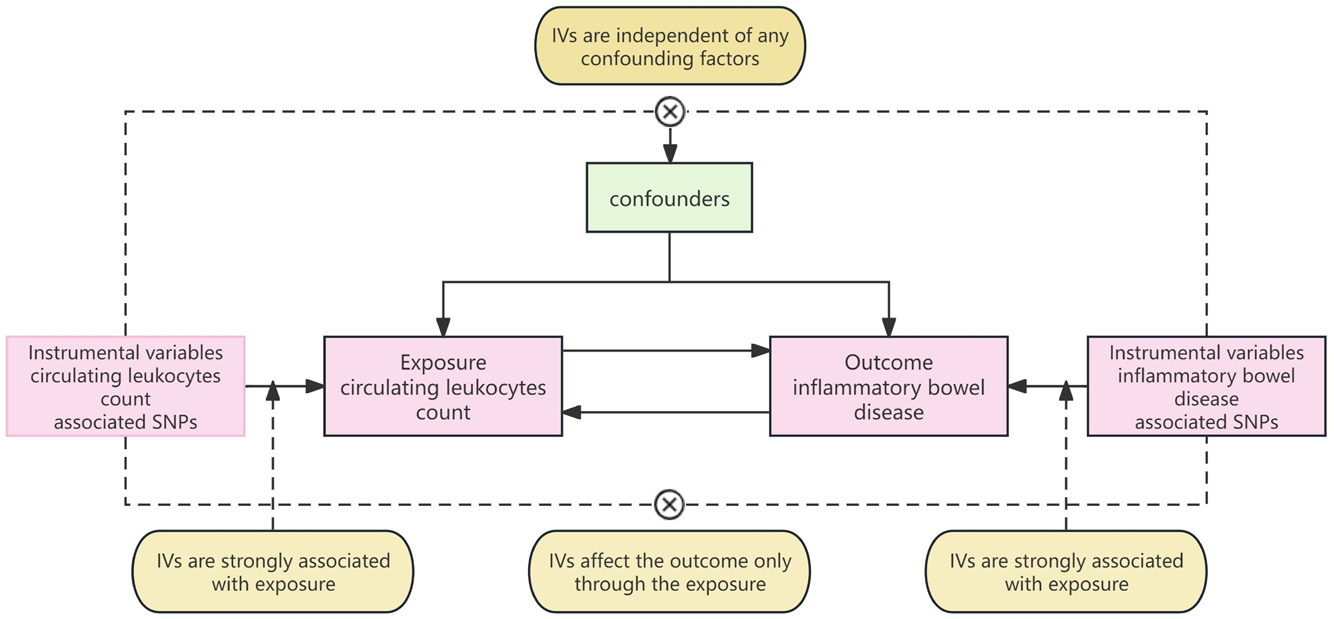
Assumptions of a MR analysis for circulating leukocytes and risk of IBD. Broken lines represent potential pleiotropic or direct causal effects between variables that would violate MR assumptions. IBD = Inflammatory bowel disease, IVs = instrumental variables, MR = Mendelian randomization.

As instrumental variables, single-nucleotide polymorphisms (SNPs) are satisfied to the 3 assumptions of MR analysis: instrumental variables (IVs) are strongly associated with exposure; IVs are independent of any confounding factors; IVs affect the outcome only through the exposure. In this study, 2-sample MR and reverse MR were used to investigate the bidirectional correlation between circulating leukocytes count and IBD (Fig. 1).

### 2.2. Exposure and outcome data sources

The 5 circulating leukocytes were monocytes, lymphocytes, eosinophils, basophils and neutrophils. The study of Astle et al^[12]^ provided the genome-wide association analysis (GWAS) data of 5 circulating leukocytes traits, which came from 172,275 people with European ancestry based on the UK Biobank and INTERVAL. Effect estimates of SNPs on the risk of IBD were evaluated with GWAS summary statistics from the Dönertaş study,^[13]^ which involved 4101 IBD cases and 480,497 control subjects of European ancestry. Summary data of UC was gathered from a recent GWAS study completed by Garcia-Etxebarria,^[14]^ which involved 208 UC cases and 935 control subjects of European ancestry. Effect estimates of SNPs on the risk of CD were evaluated with GWAS summary statistics from the Liu study,^[15]^ which involved 5956 CD cases and 14,927 control subjects of European ancestry (Table S1, Supplemental Digital Content, http://links.lww.com/MD/O596).

### 2.3. Selection of instrumental variables

In constructing the IVs, genetic variants of the SNPs (*P* < 5E−08) significantly associated with circulating leukocytes count were selected. To ensure independence among the IVs, variants with potential linkage disequilibrium were removed (kb = 10,000, *r*^2^ < 0.001). In addition, SNPs significantly associated with IBD (UC and CD) (*P* < 5E−05) were excluded to improve the validity of instrumental variables. To harmonize the IVs, the maximum minor allele frequency threshold for aligning palindromic SNPs is set to 0.3. We calculated the *F* statistic [*F* = ((*R*^2^/(1 − *R*^2^)) × ((N − *K* − 1)/*K*)] of each SNP. Specifically, N represents the sample size of the exposure data, and *R*^2^ represents the explained variance of the genetic instruments. Finally, we eliminated the SNPs identified as weak IVs (*F* < 10). In reverse MR, the filtering method was the same as in forward MR. Only for UC, we used a threshold of 5 × 10^−6^ due to fewer eligible SNPs.

### 2.4. Statistical analysis

MR Analysis was carried out mainly using the “Two-sample MR” toolkit using R Studio in R 3.5.3 software. In our study, a bidirectional MR analysis of circulating leukocytes count and IBD (UC and CD) was performed. Inverse variance weighting (IVW) was used as the primary MR analysis method, and provided a concise estimate of potential heterogeneity. Therefore, in the presence of heterogeneity, the random effects IVW model was implemented. Otherwise, the fixed effects IVW model was used. In order to strengthen the robustness of the findings, if causality was significantly present in the IVW approach, and no contradictory results were found in the sensitivity analysis, the significant causal effect of exposure on the outcome was considered.

Because the IVW estimate was based on the no measurement error (NOME) assumption, we detected the pleiotropy and heterogeneity to test sensitivity. Pleiotropy was assessed using the MR Egger and MR pleiotropy residual sum and outlier (MR-PRESSO) methods to ensure the availability of data. The outliers identified by MR-PRESSO method were deleted and IVW analysis was performed. Cochran *Q* test was used for heterogeneity analysis, and leave-one-out was used to detect IV outliers substantially influencing causal effects, which showed that the pathogenic effect of exposure on the outcome was not controlled by a single SNP.

## 3. Results

### 3.1. MR analysis of circulating leukocytes count and IBD

**Figure 2.**
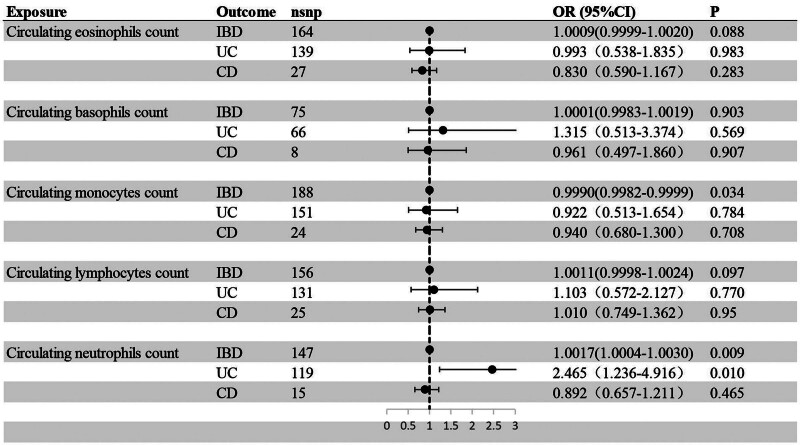
MR estimated of the association between circulating leukocytes count and risk of IBD. CI = confidence interval, IBD = Inflammatory bowel disease, MR = Mendelian randomization, OR = odds ratio.

The detailed causal estimates of circulating leukocytes count on IBD risk were summarized in Table S2, Supplemental Digital Content (http://links.lww.com/MD/O598). Across 5 major leukocytes subtypes, monocytes, lymphocytes, eosinophils, and basophils count were not significantly associated with IBD. However, circulating neutrophils count was positively associated with IBD risk using IVW method [odds ratio (OR) = 1.002; 95% confidence interval (CI)  = 1.001–1.003; *P* = .009]. Furthermore, our results showed that in subtypes of IBD, circulating neutrophils count was positively associated with UC risk, with a significant causal effect [OR = 2.465; 95% CI = 1.236–4.916; *P* = .01] (Fig. 2).

**Figure 3.**
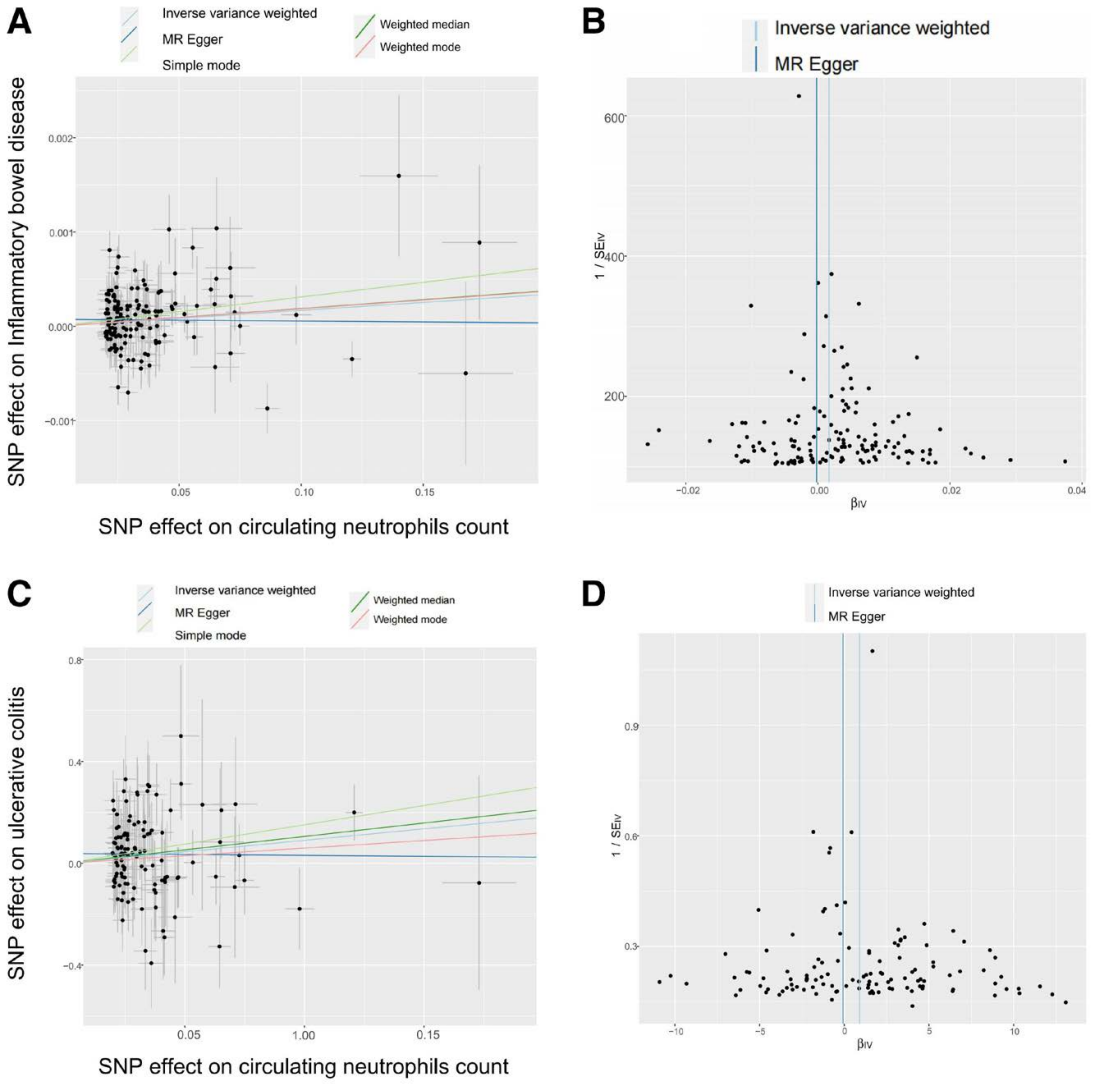
MR plots for reviewing relationship between circulating leukocytes count and IBD using MR. (A) Scatter plot of SNP effects on circulating leukocytes count versus IBD; (B) Funnel plot to assess heterogeneity of circulating leukocytes count versus IBD. (C) Scatter plot of SNP effects on circulating leukocytes count versus UC; (D) Funnel plot to assess heterogeneity of circulating leukocytes count versus UC. IBD = Inflammatory bowel disease, MR = Mendelian randomization, SNP = single-nucleotide polymorphism, UC = ulcerative colitis.

In this MR analysis, Egger-intercept *P*-values were not statistically significant, indicating that horizontal pleiotropy did not exist (Table S3, Supplemental Digital Content, http://links.lww.com/MD/O599). Heterogeneity analysis of IVs was assessed via the Cochran *Q* test, and there was no heterogeneity among IVs (Table S3, Supplemental Digital Content, http://links.lww.com/MD/O599). In addition, the results of the leave-one-out analysis showed that the causal relationship between circulating neutrophils count and IBD and UC remained stable and were not affected by a single SNP (Table S4, Supplemental Digital Content, http://links.lww.com/MD/O600, Fig. S1, Supplemental Digital Content, http://links.lww.com/MD/O604), which were supported by Scatter (Fig. 3A and C) and Funnel plots (Fig. 3B and D) of MR tests. Taken together, these data strongly supported the causal relationship between circulating neutrophils and IBD, especially UC.

### 3.2. Reverse MR analysis assessing the effect of IBD on circulating leukocytes count

**Figure 4.**
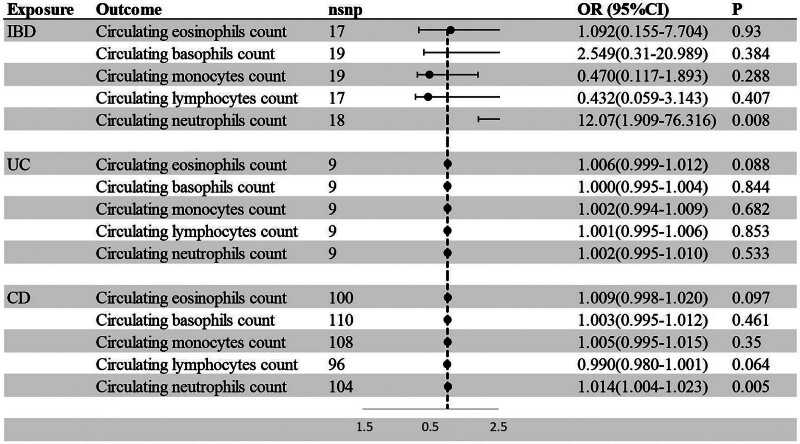
MR estimated of the association between IBD and circulating leukocytes count. CI = confidence interval, IBD = Inflammatory bowel disease, MR = Mendelian randomization, OR = odds ratio.

In order to investigate the origin of the observed correlations between circulating leukocytes count and IBD, we used IBD-associated variants as IVs in the reverse direction. The detailed causal estimates of IBD on circulating leukocytes count were summarized in Table S5, Supplemental Digital Content (http://links.lww.com/MD/O601). According to our MR analysis, IBD, including UC and CD were not significantly associated with lymphocytes, basophils, eosinophils, or monocytes count. However, we founded that IBD susceptibility was robustly associated with higher circulating neutrophils count using IVW method [OR = 12.07; 95% CI = 1.909–76.316; *P* = .008]. Between the IBD subtypes, we also found suggestive evidence that CD was positively associated with circulating neutrophils count [OR = 2.465; 95% CI = 1.236–4.916; *P* = .01] (Fig. 4).

**Figure 5.**
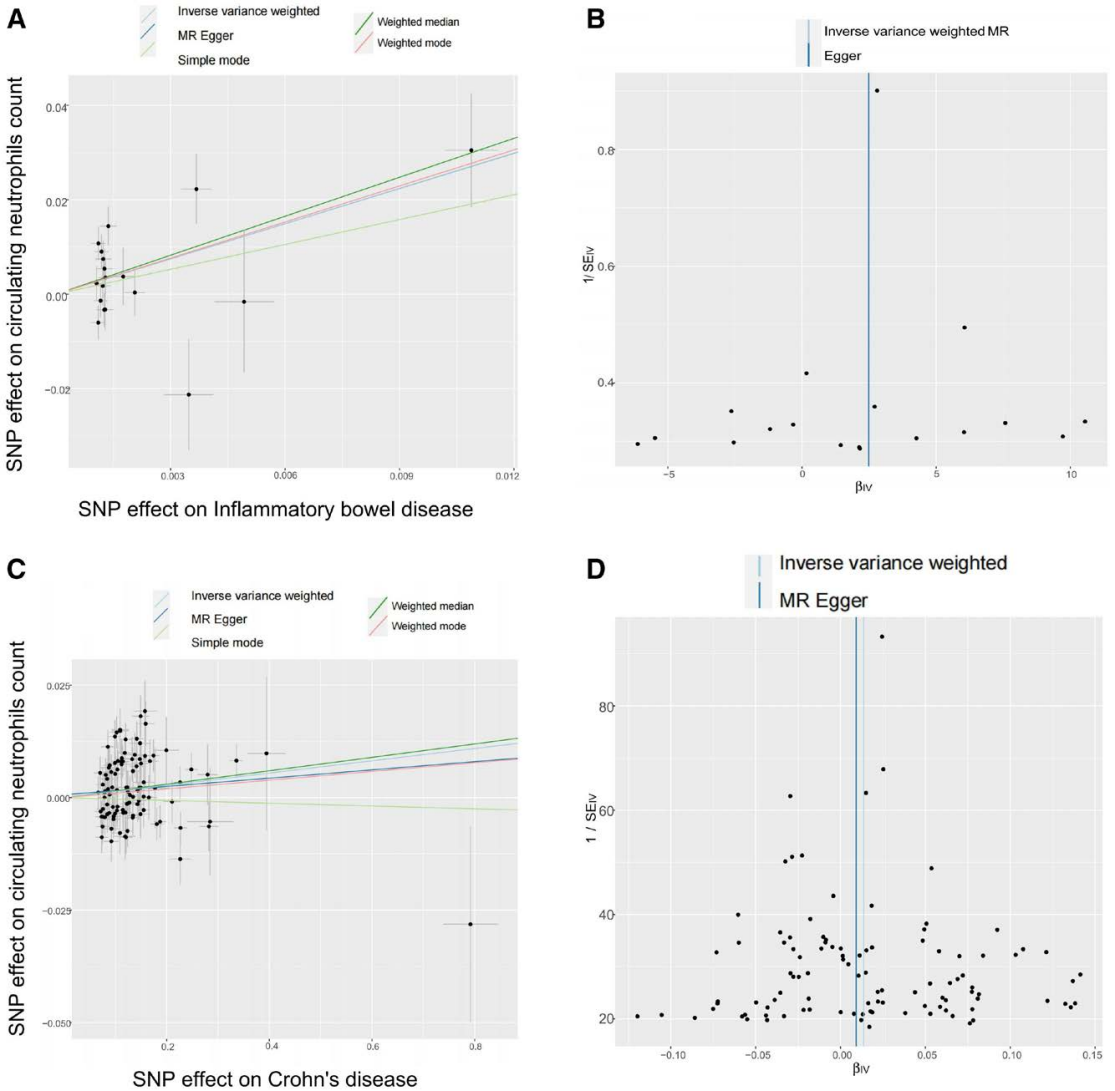
MR plots for reviewing relationship between IBD and circulating leukocytes count using MR. (A) Scatter plot of SNP effects on IBD and circulating leukocytes count; (B) Funnel plot to assess heterogeneity of IBD and circulating leukocytes count; (C) Scatter plot of SNP effects on CD and circulating leukocytes count; (D) Funnel plot to assess heterogeneity of CD and circulating leukocytes count. IBD = Inflammatory bowel disease, MR = Mendelian randomization, SNP = single-nucleotide polymorphism, UC = ulcerative colitis.

In this MR Analysis, we further detected the pleiotropy and heterogeneity to test sensitivity. The test results showed that there was no heterogeneity and horizontal pleiotropy in data used (Table S6, Supplemental Digital Content, http://links.lww.com/MD/O602). In addition, leave-one-out analysis did not give evidence of SNPs disproportionally affecting the effect estimates of IBD, CD and circulating neutrophils count (Table S7, Supplemental Digital Content, http://links.lww.com/MD/O603, Fig. S2, Supplemental Digital Content, http://links.lww.com/MD/O605). Scatter and funnel plots of MR tests for IBD, CD and circulating neutrophils count also showed consistent trends (Fig. 5A–D).

## 4. Discussion

Clinical studies are difficult to draw causal inferences due to reverse causation and confusion. In comparison, the MR approach, which uses genetic variants robustly associated with the exposure as IVs, can be used to infer causality while controlling for various sources of confounder. To understand the causal relationship between circulating leukocytes count and IBD including UC and CD, we used publicly aggregated GWAS data for 2-way MR analysis. The forward MR results showed that the increased levels of circulating neutrophils count increase the risk of IBD. For IBD subtypes, increased levels of circulating neutrophils count increased the risk of UC. In our reverse MR analysis, we first found evidence that the risk for IBD and CD were positively associated with circulating neutrophils count.

Neutrophils are derived from a common myeloid progenitor present in the bone marrow and extramedullary tissues, which represent 50% to 70% of the total pool of circulating leukocytes in humans.^[16]^ Meanwhile, it is now recognized that neutrophils play a role in the initiation, modulation and resolution phases of inflammation.^[17]^ Neutrophils can pass through the capillary wall through deformation and are quickly recruited to sites of infection or inflammation. They are the first to infiltrate the gut mucosa to maintain the inflammatory response.^[18]^ A recent study showed that neutrophils could release a series of cytokines and chemokines to enhance the inflammatory response of the gut.^[19]^ Neutrophils participate in the elimination of microorganisms through phagocytosis, degranulation, the generation of reactive oxygen species, and the release of neutrophils extracellular traps (NETs).^[20]^ Neutrophils could produce an excess of reactive oxygen species to induce excessive oxidation reaction, and meet the condition of oxidative stress. Next, oxidative stress maintains inflammation in the gut mucosa by activating redox-sensitive signaling pathways and transcription factors.^[21]^ A clinical study found that infliximab, a tumor necrosis factor inhibitor, significantly reduced the circulating leukocytes and neutrophil counts in patients with IBD, thereby improving the intestinal inflammatory response of patients.^[22]^ These findings will support the broader use of antineutrophilic therapy in the management of UC and IBD. Therefore, inhibit ion of neutrophils can reduce the symptoms and inflammation of IBD and will be the main strategy for the treatment of IBD in the future. In addition, while some studies have indicated a potential correlation between these immune cells (monocytes, lymphocytes, eosinophils, basophils) and IBD,^[23–25]^ but our results have not yet shown a causal relationship among them. Perhaps in the future, as research deepens, the relationship between them will be further revealed. Our MR result suggested that circulating neutrophils count may increase risk of UC. NET is a network of structures protruding from the membrane of activated neutrophils, including chromatin, DNA, and antimicrobial peptides. Neutrophils can sustain intestinal mucosal inflammation and damage in UC patients by forming NETs.^[26]^ A recent study found higher plasma NETs levels and presence of NETs in colon tissues in patients with active UC, whose mechanism involved impairing intestinal barrier function, exacerbating colonic tissue damage and driving thrombotic tendency.^[27]^ Consistent with our research findings, persistent abnormal neutrophils infiltration in the colonic tissue elevates intestinal and systemic neutrophil activity, and participates in immune responses via NETs. However, excessive NETs perforated and degraded tissue cell membranes, leading to the cell death, local tissue damage, and disruption of the intestinal mucosal barrier, thereby increasing intestinal inflammation and exacerbating UC.^[28]^ Of note, the NETs could also accelerate the transformation of colitis into cancer.^[29]^ Similarly, an animal study showed that inhibiting NETs release in mice attenuated colitis and colitis-related tumorigenesis.^[27]^ Upregulation of c-MET receptor tyrosine kinase in neutrophils could also stimulated pro-inflammatory phenotypes and promoted the development of UC.^[30]^ On the other hand, a randomized controlled trial showed that all neutrophils-related markers (neutrophils gelatinase B-associated lipocalin and matrix metalloproteinase-9, catholiciding LL-37 and chitinase 3-like 1) were significantly higher in active UC patients compared to healthy controls.^[31]^ In summary, elevated circulating neutrophils may be a potential cause of UC. Our MR study indicated that the high circulating neutrophils count has a significant statistical difference in the risk of UC, but has no significant impact on CD. This aligns with the viewpoints presented in recently published high-quality articles.^[32]^ The contribution of neutrophils to disease progression differs between patients with UC and CD. The correlation between neutrophils infiltration and the severity of UC is more closely related than that between neutrophils and CD.^[33]^ Although neutrophils may be involved in the occurrence and development of CD, the pathogenesis of CD is more related to immune dysfunction of CD4 + T cells.^[34]^

Our MR study also indicated that IBD, specifically CD, is a risk factor for elevated circulating neutrophil counts. All our discoveries suggest a bidirectional causal relationship between circulating neutrophils count and IBD (including UC and CD), which may be attributed to the combined causal effects between circulating neutrophils count and both UC and CD. IBD is a chronic recurrent disease of the gastrointestinal tract with abdominal pain, diarrhea, mucous with blood as the main clinical manifestations. A clinical study has shown that patients with IBD have a wide range of genetic disorders in their blood compared to healthy people. Dysregulated mRNAs and miRNAs are mainly involved in innate immunity, especially neutrophils activation related pathways.^[35]^ This study further confirms that IBD can lead to overactivation of neutrophils, aligning with our initial analysis. CD, one of the IBD subtypes, was a chronic inflammatory disease of the gastrointestinal tract, with increasing incidence worldwide. The main pathological feature of CD is an infiltration of polymorphonuclear neutrophils and mononuclear cells into the affected part of the intestine. A study found an imbalance in the levels of pro-inflammatory and anti-inflammatory cytokines occurred in CD, which increased the production of neutrophils, chemokines and interleukin-8.^[36]^ Meanwhile, another study showed the NETs density increased with increasing histopathological severity of CD,^[37]^ indicating that circulating neutrophils count increased with the severity of CD. On the other hand, increased mucosal permeability might contribute to the pathogenesis of the intestinal lesions in CD by means of increased absorption of formyl-methionyl-leucyl-phenylalanine and other gut-derived bacterial products.^[38]^ A meta-analysis showed that peripheral neutrophil to lymphocyte ratio was significantly higher in peripheral blood of patients with active CD than in healthy people, and could serve as an important biomarker of CD severity.^[39]^ Indeed, in patients with CD, impaired neutrophils function leads to reduced oxidative and phagocytic activities, resulting in delayed or incomplete bacterial clearance. The persistent presence of bacterial antigens in the tissue triggers an upregulation of intestinal adaptive immune responses and the formation of granulomas, causing an elevated reactivity of circulating neutrophils count. In summary, CD may be a potential cause of elevated circulating neutrophils.

We are the first to confirm a causal relationship between circulating neutrophils count and IBD from a genetic perspective, and emphasized the important role of neutrophils in IBD. By employing bidirectional MR, we confirmed the directionality and independence of this causal relationship. Our study used European population-based GWAS data to ensure the precision, and performed rigorous IVs screening and sensitivity analysis to ensure the reliability of the results. However, the limitations of this research should be acknowledged as well. There were some differences in the GWAS data we used, particularly UC, which may affect the analysis results to some extent. Additionally, our study results pertained primarily to European populations, and generalization to other populations warrants further investigation. The GWAS data used were derived from European populations and generalization to other populations warrants further investigation. The underlying mechanism between circulating neutrophils count and IBD (UC and CD) needs to be confirmed by further studies. In addition, further study of the causal relationship between neutrophil subsets and IBD is also critical.

## 5. Conclusions

This MR Study provides strong genetic evidence for a causal relationship between the genetically predicted increase in circulating neutrophils count and the risk of IBD (UC and CD). This finding stresses the need for further exploring physiological functions of neutrophils in order to develop effective strategies against IBD.

## Author contributions

**Conceptualization:** Zhibin Wang, Tangyou Mao.

**Data curation:** Chengtao Liang, Junxiang Li.

**Funding acquisition:** Junxiang Li, Tangyou Mao.

**Investigation:** Chengtao Liang, Yuhe Mai, Qiuhong Dai, Yali Yuan, Muyuan Wang, Yuyue Liu, Wenji Zhang, Yitong Li, Xinyu Lu, Zhengdao Lin.

**Methodology:** Zhibin Wang, Junxiang Li.

**Project administration:** Yitong Li.

**Resources:** Tangyou Mao.

**Software:** Chengtao Liang, Yuhe Mai, Qiuhong Dai, Muyuan Wang, Xinyu Lu, Zhengdao Lin.

**Supervision:** Wenji Zhang.

**Validation:** Yali Yuan, Yuyue Liu.

**Writing – original draft:** Chengtao Liang, Yuhe Mai, Qiuhong Dai.

**Writing – review & editing:** Zhibin Wang, Junxiang Li, Tangyou Mao.

## Supplementary Material


