# Supplementary material for: Mendelian randomization study of circulating leukocytes counts reveals causal associations with inflammatory bowel disease
Source: Medicine (Baltimore). 2025 Mar 28;104(13):e41969. doi: 10.1097/MD.0000000000041969 (PMC11957634; doi:10.1097/MD.0000000000041969)
Supplement: Supplementary file 5 [file medi-104-e41969-s005.pdf]

Mendelian randomization study of circulating leukocytes counts reveals causal associations with inflammatory bowel disease

Chengtao Liang[1§], Qiuhong Dai[2§], Yuhe Mai[1§], Yali Yuan[1], Muyuan Wang[1], Yuyue Liu, Wenji Zhang[1], Yitong Li[1], Xinyu Lu[1], Zhengdao Lin[1], Zhibin Wang[1*], Junxiang Li[1§], and Tangyou Mao[1*]

Supplementary information

Supplementary Figure 1: Leave-one-out sensitivity analysis of the significant causal effect of circulating leukocytes count on IBD and UC.

IBD, inflammatory bowel disease; UC, ulcerative colitis; CD, Crohn's disease.

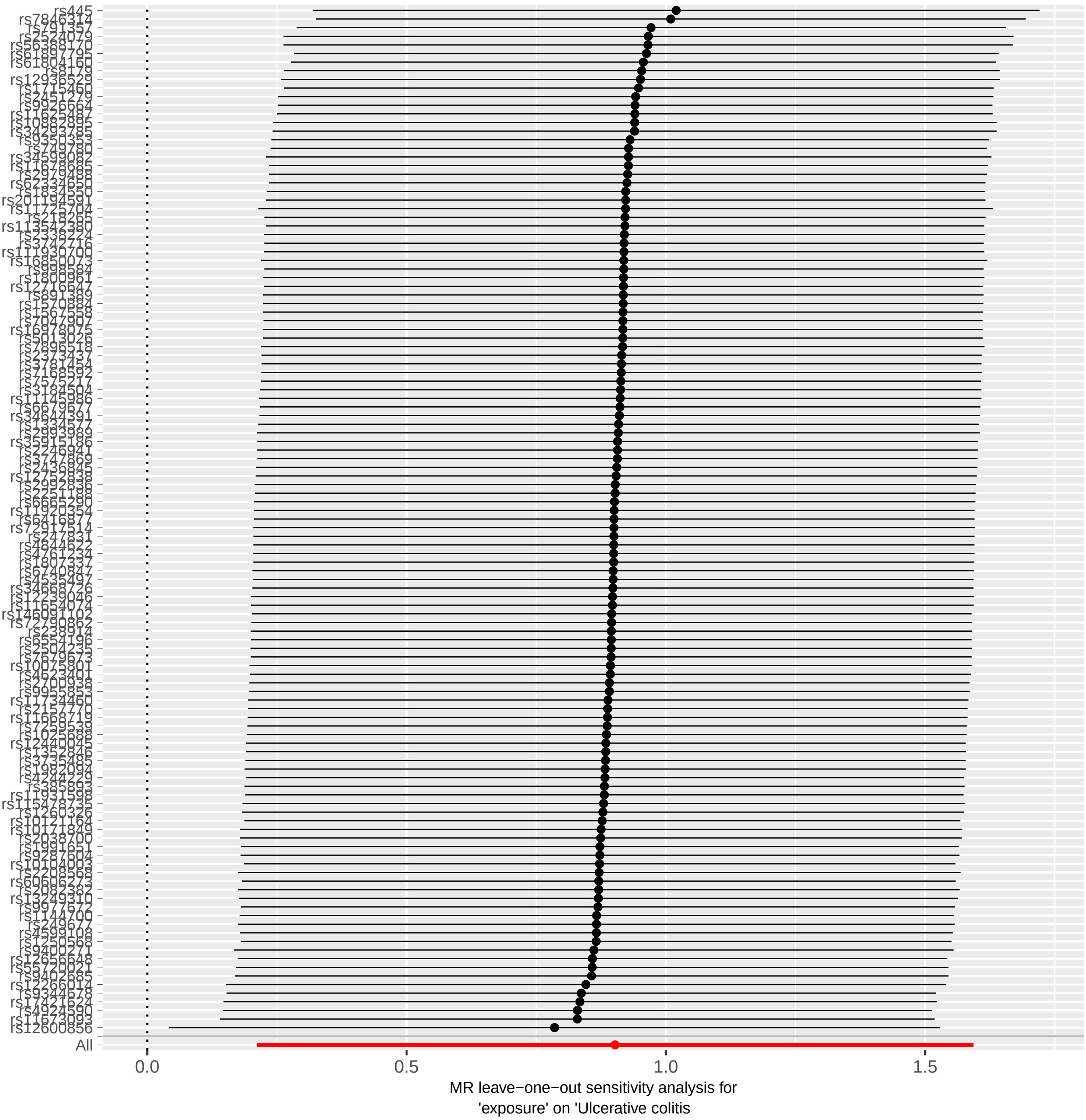
rs445
rs12600856
All
0.0
0.5
1.0
1.5
MR leave−one−out sensitivity analysis for
'exposure' on 'Ulcerative colitis

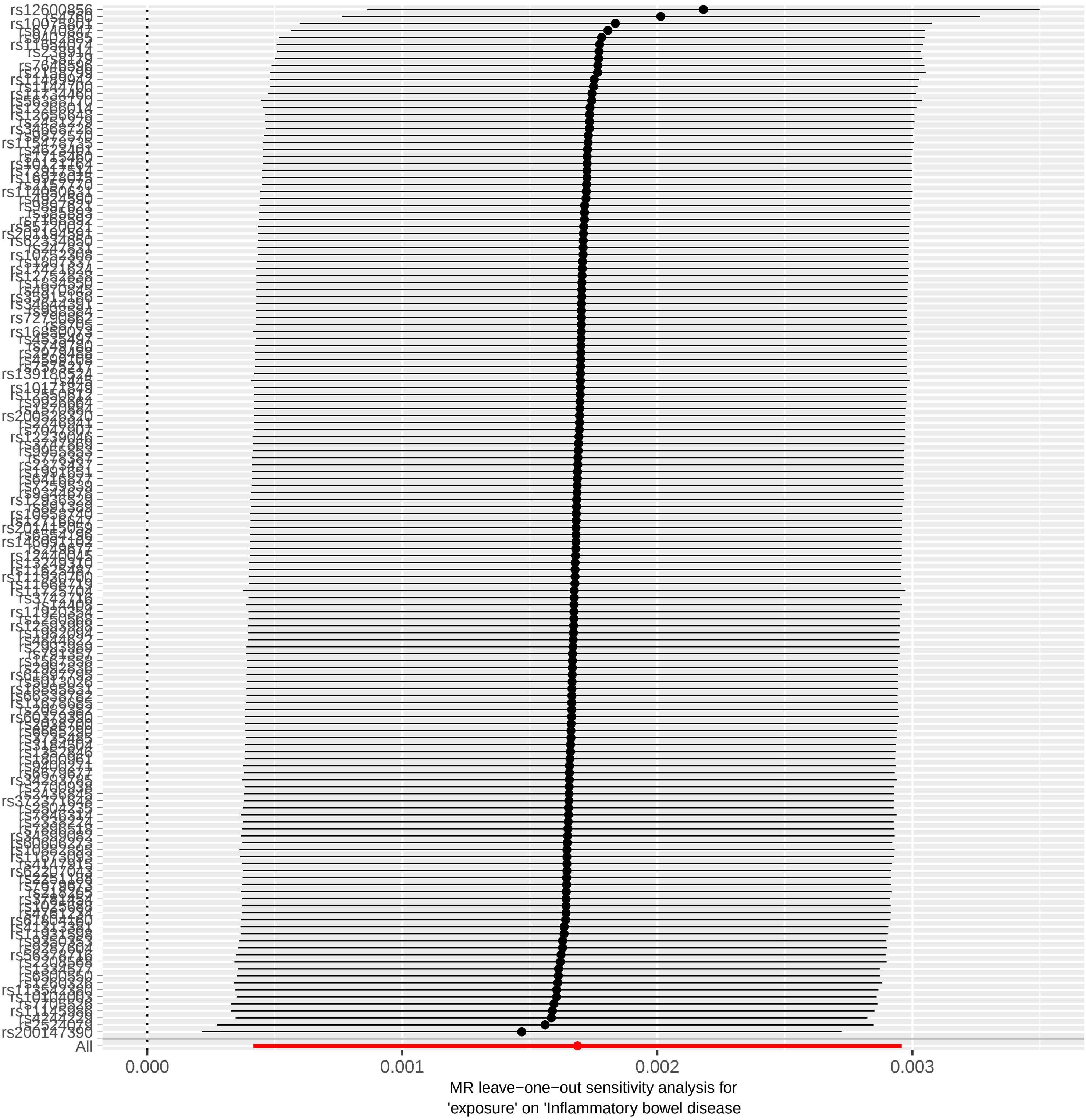
rs12600856
rs200147390
All
0.000
0.001
0.002
0.003
MR leave−one−out sensitivity analysis for
'exposure' on 'Inflammatory bowel disease
