# Supplementary material for: Mendelian randomization study of circulating leukocytes counts reveals causal associations with inflammatory bowel disease
Source: Medicine (Baltimore). 2025 Mar 28;104(13):e41969. doi: 10.1097/MD.0000000000041969 (PMC11957634; doi:10.1097/MD.0000000000041969)
Supplement: Supplementary file 9 [file medi-104-e41969-s009.pdf]

Mendelian randomization study of circulating leukocytes counts reveals causal associations with inflammatory bowel disease

Chengtao Liang[1§], Qiuhong Dai[2§], Yuhe Mai[1§], Yali Yuan[1], Muyuan Wang[1], Yuyue Liu, Wenji Zhang[1], Yitong Li[1], Xinyu Lu[1], Zhengdao Lin[1], Zhibin Wang[1*], Junxiang Li[1§], and Tangyou Mao[1*]

Supplementary Figure 2: Leave-one-out sensitivity analysis of the significant causal effect of IBD and UC on circulating leukocytes count .

IBD, inflammatory bowel disease; UC, ulcerative colitis; CD, Crohn's disease.

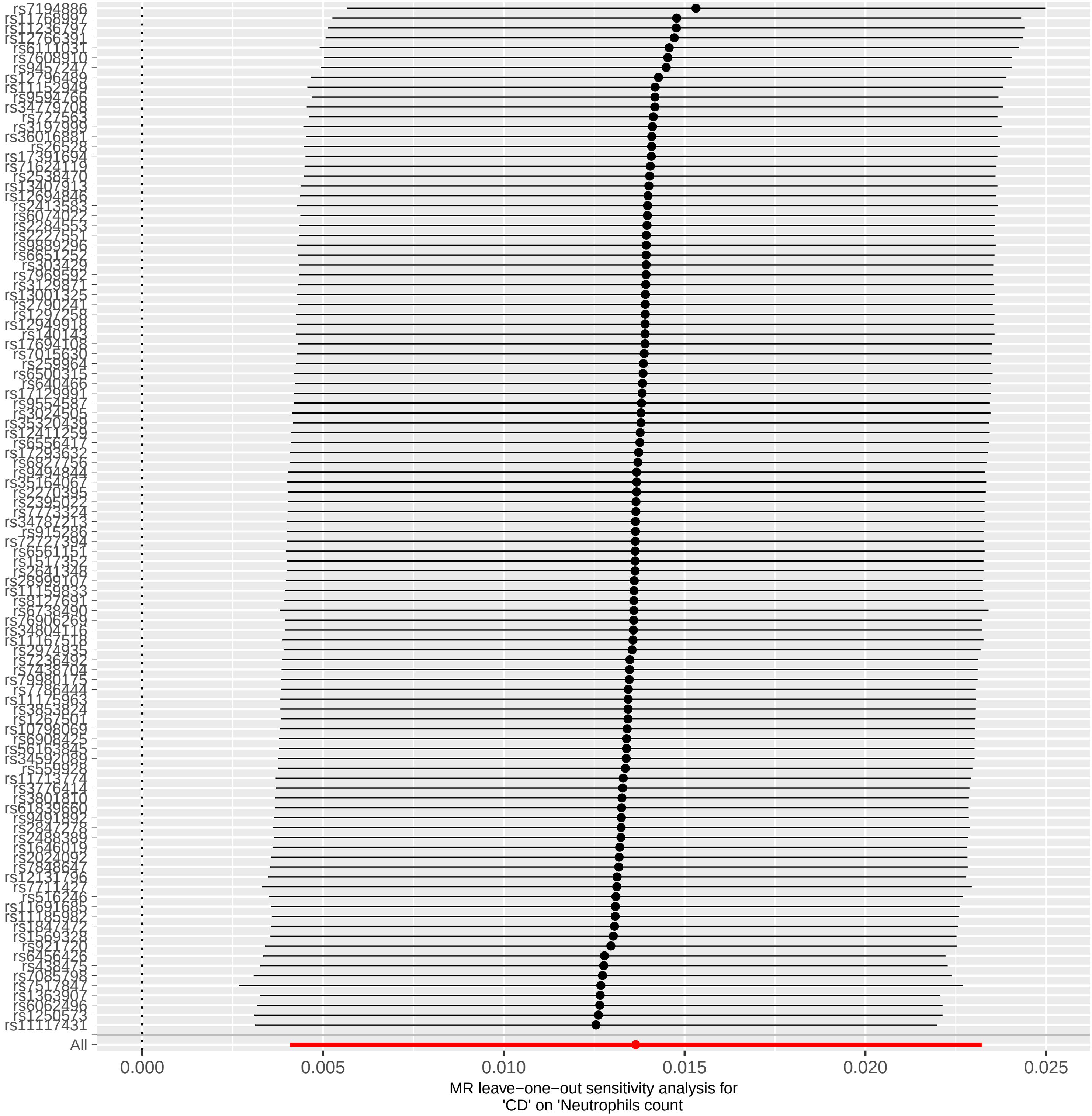
All
0.000
0.005
0.010
0.015
0.020
0.025
MR leave−one−out sensitivity analysis for
'CD' on 'Neutrophils count'

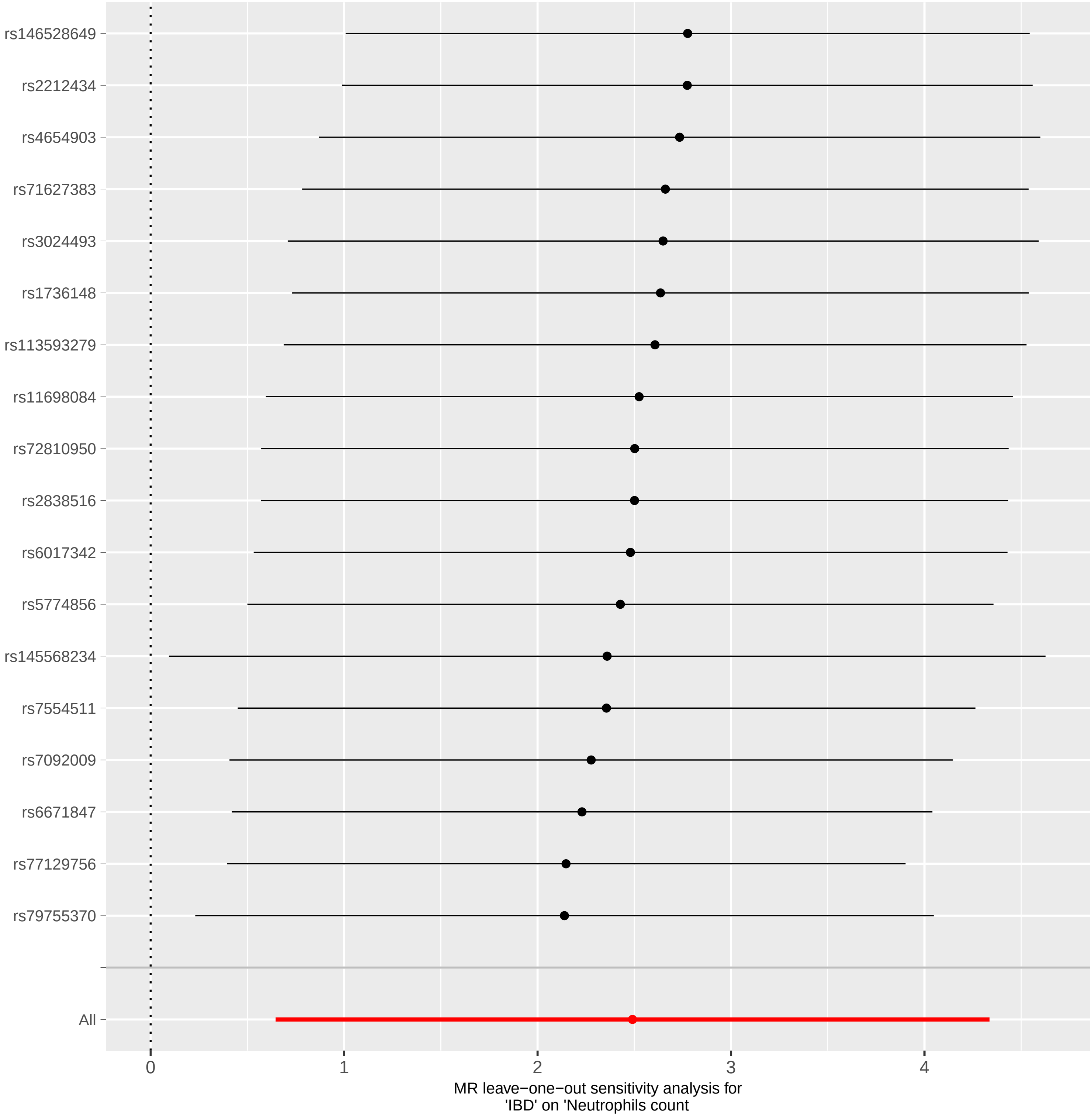
rs146528649
rs2212434
rs4654903
rs71627383
rs3024493
rs1736148
rs113593279
rs11698084
rs72810950
rs2838516
rs6017342
rs5774856
rs145568234
rs7554511
rs7092009
rs6671847
rs77129756
rs79755370
All
0
1
2
3
4
MR leave−one−out sensitivity analysis for
'IBD' on 'Neutrophils count
